# Lost in translation: an overinterpretation of the acute effects of cannabinoids

**DOI:** 10.1038/s41398-019-0669-1

**Published:** 2019-12-18

**Authors:** James G. Holland, Ciara A. Torres

**Affiliations:** 1 Columbia University, 901 Northwest Corner Building, 550 West 120th Street, New York, NY 10027 USA; 2 Columbia University, 116th St & Broadway, New York, NY 10027 USA

**Keywords:** Human behaviour, Scientific community

## Dear editor,

As legislation in the United States and elsewhere changes to allow more people to use cannabis and its derivatives, it is imperative that we understand the full spectrum of the effects of its psychoactive compounds in humans. With this in mind, we read the article by Morgan et al.^1^, which aimed to do just that, with great interest. The authors administered tetrahydrocannabinol (THC) and cannabidiol (CBD) to participants with varying levels of schizotypy. Participants then completed a series of tests to assess a range of cognitive domains, as well as psychotic symptoms. The researchers conclude that participants showed “cognitive impairments” after THC administration. However, a number of limitations preclude translation of these findings to the use of cannabis in its relevant milieu.

First, the authors did not test the effects of cannabis itself; participants inhaled vaporized ethanol solutions containing pure THC, CBD, or a 1:2 THC:CBD mix. Cannabis is complex,containing dozens of largely uncharacterized psychoactive compounds^2^. One or two of these compounds administered in isolation do not recreate the effect of cannabis on the brain. Future studies could examine established cannabis strains with the desired cannabinoid concentrations. This will ensure that we gain knowledge about cannabinoid effects that represent those that might be experienced by most users in the general public.

Second, the doses that were administered (8 mg of THC, 16 mg of CBD) do not reflect those typically used in a single sitting (32.24 mg of THC and 0.56 mg of CBD in an entire joint^3^). Rather, the authors administered doses that produced “psychotic-like symptoms and memory impairment”. In other words, they used what would induce the desired effects rather than what relates to common cannabis use patterns. This renders their findings irrelevant to most cannabis use. Moreover, many drugs result in negative health outcomes at high doses, but are relatively harmless or even medicinal at low doses^4^.

Finally, the participants tested did not represent a normal population in several ways. Participants scored in either the top or bottom quartiles for schizotypy within a group of cannabis users and smoked either 1–24 or 25+ days per month. These group subdivisions, especially in terms of the smoking frequency, seem arbitrary. Because no cannabis-abstinent or non-schizotypal (from a cannabis-abstinent population) control was examined, it is not possible to rule out participants’ history of cannabis use itself as a confound. Even with these confounds, the results were not compared with a normative dataset, despite the use of standardized tests for which this is available (e.g., Psychotomimetic States Inventory^5^). That these data demonstrate “a pro-psychotic effect of THC” might, at best, be valid only for this specific population.

We recognize that cannabinoid administration can induce acute psychological changes, and albeit rare, even psychotic-like states. Our concern is that the paper by Morgan et al.^1^ does not address the effects of cannabinoids in a way that is focused on or relevant to most cannabis use. In order to do this, participant recruitment and both drug dose and route of administration should be chosen with cannabis use norms in mind. Without this, data do not have much significance outside of the narrow experimental setting in which they were obtained. Moreover, comparison of cognitive scores to normative data to determine clinical relevance is at the core of clinical neuropsychology. This must also be adhered to when studying the effects of drug exposure. Not doing so could not only lead to overinterpretations in the literature but also hinder the ability of clinicians, policymakers, and the public to engage in scientifically sound discussions about cannabis and its related effects.
